# Supplementary material for: Intracellular Delivery of siRNAs Targeting AKT and ERBB2 Genes Enhances Chemosensitization of Breast Cancer Cells in a Culture and Animal Model
Source: Pharmaceutics. 2019 Sep 3;11(9):458. doi: 10.3390/pharmaceutics11090458 (PMC6781320; doi:10.3390/pharmaceutics11090458)
Supplement: Supplementary file 1 [file pharmaceutics-11-00458-s001.pdf]

pharmaceutics

MDPI

Supplementary Materials: Intracellular Delivery of siRNAs Targeting AKT and ERBB2 Genes Enhances Chemosensitization of Breast Cancer Cells in a Culture and Animal Model

Tahereh Fatemian, Hamid Reza Moghimi and Ezharul Hoque Chowdhury

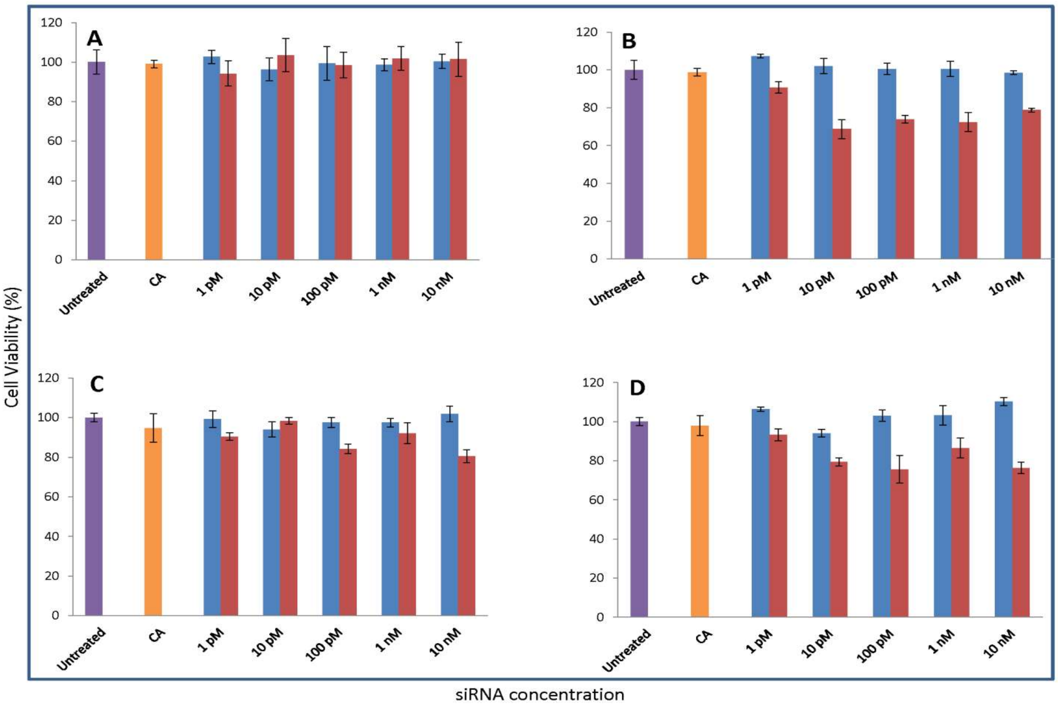

Figure S1. Cell viability assessment in 4T1 cells treated with siRNA loaded carbonate apatite. Nanoparticles were formed by using 4 mM of Calcium together with 1 pM to 10 nM of each siRNA (**A**: AKT, **B**: ERBB2, **C**:MAPK, **D**: ROS1). Two days after treatment, MTT assay was performed. Values are presented as cell viability (%) compared to untreated cells. ●Free siRNA ●CA/siRNA.

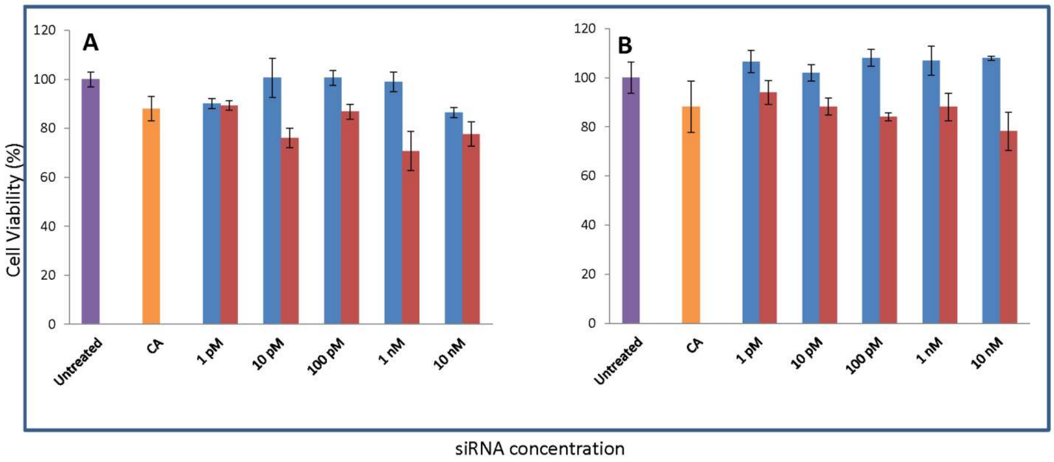

Figure S2. Carbonate apatite facilitated delivery of ERBB2 (**A**) and ROS1 (**B**) siRNA to MCF-7 cells. With 4 mM of $CaCl_2$ and various concentrations of siRNA (1 pM to 10 nM), cells were treated and MTT assay was performed two days after treatment. Values are presented as cell viability (%) compared to untreated cells. ●free siRNA ● CA/siRNA.

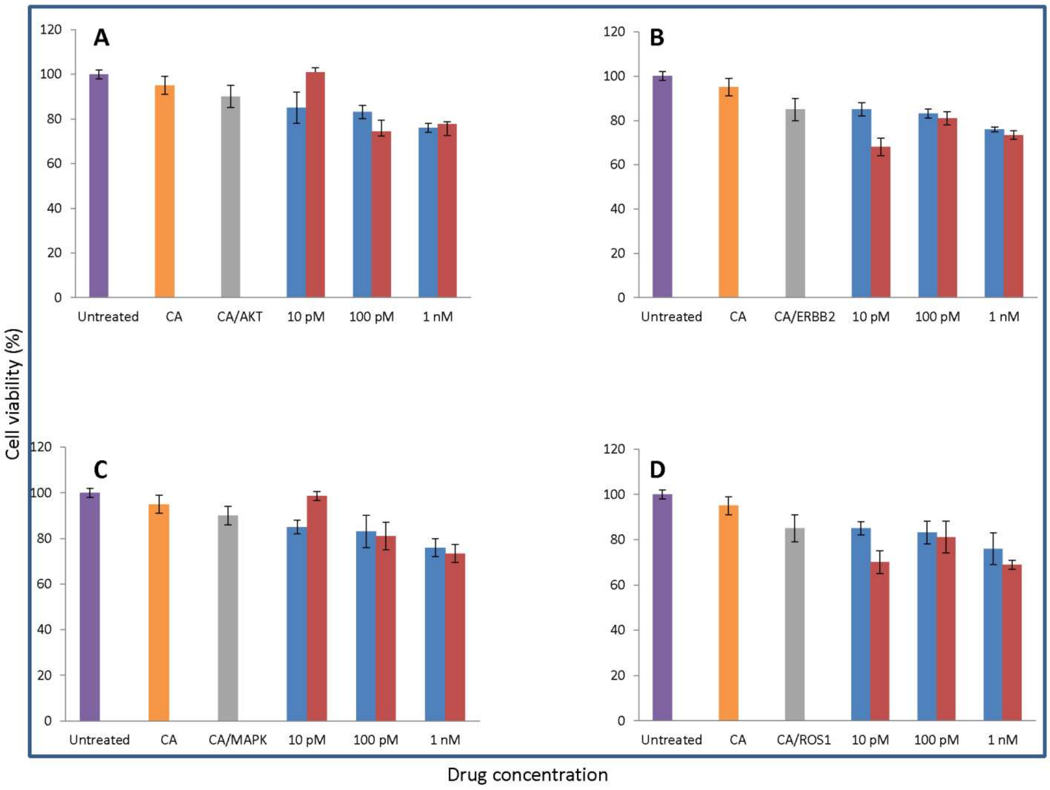

Figure S3. Effect of single pathway silencing on cytotoxicity of paclitaxel on 4T1 cells. By incorporation of 3 mM Ca, 1 pM AKT (**A**), ERBB2 (**B**), MAPK (**C**) and ROS1 (**D**) siRNA together with 10 pM, 100 pM and 1 nM paclitaxel into apatite structure, cells were treated. Two days post treatment, MTT assay was conducted to produce results of cell viability (%) compared to untreated cells. ● CA/Pac, ● CA/siRNA/Pac.

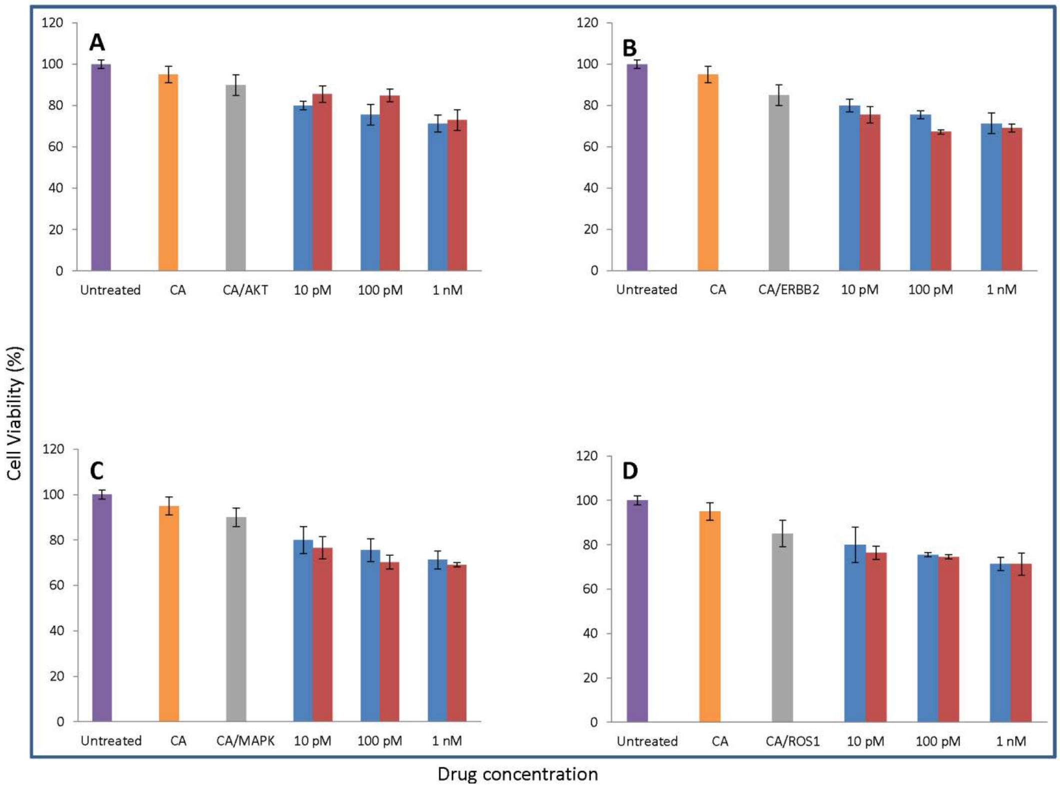

Figure S4. Effect of single pathway silencing on cytotoxicity of docetaxel on 4T1 cells. By incorporation of 3 mM Ca, 1 pM AKT (**A**), ERBB2 (**B**), MAPK (**C**) and ROS1 (**D**) siRNA together with 10 pM, 100 pM and 1 nM docetaxel into apatite structure, cells were treated. Two days post treatment, MTT assay was conducted to produce results of cell viability (%) compared to untreated cells. ● CA/Doc, ● CA/siRNA/Doc.

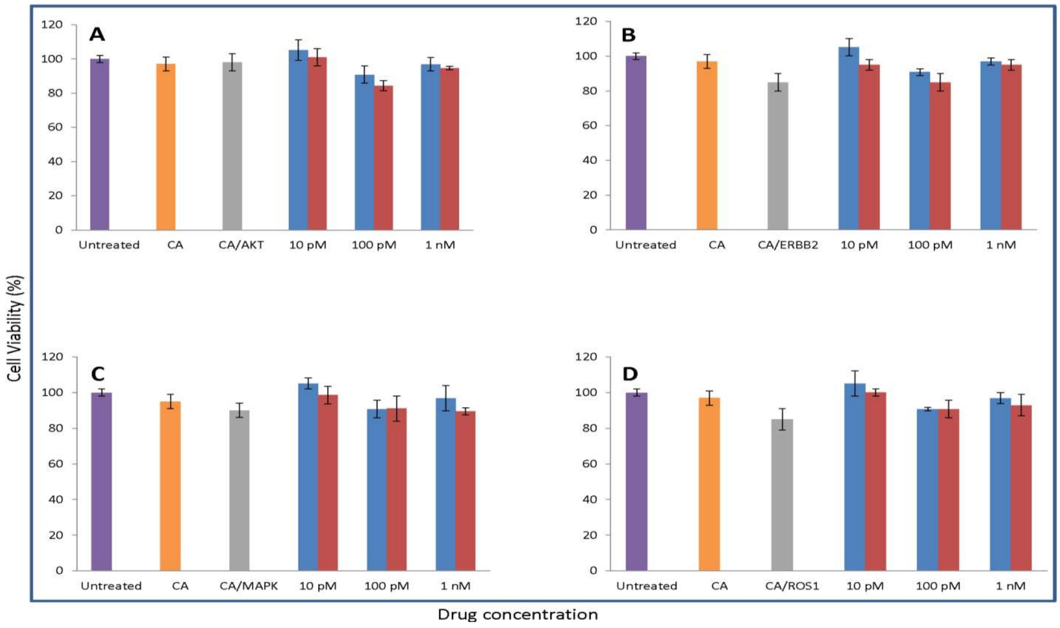

Figure S5. Effect of single pathway silencing on cytotoxicity of mitomycin C on 4T1 cells. By incorporation of 3 mM Ca, 1 pM AKT (**A**), ERBB2 (**B**), MAPK (**C**) and ROS1 (**D**) siRNA together with 10 pM, 100 pM and 1 nM Mitomycin C into apatite structure, cells were treated. Two days post treatment, MTT assay was conducted to produce results of cell viability (%) compared to untreated cells. ● CA/Mito, ● CA/siRNA/Mito.

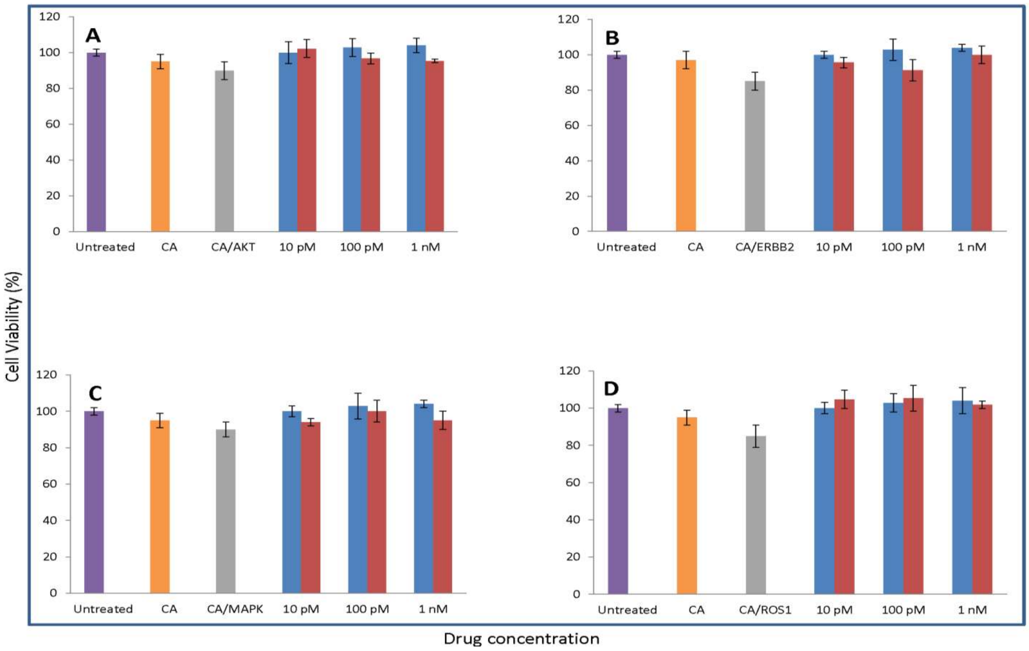

Figure S6. Effect of single pathway silencing on cytotoxicity of topotecan on 4T1 cells. By incorporation of 3 mM Ca, 1 pM AKT (**A**), ERBB2 (**B**), MAPK (**C**) and ROS1 (**D**) siRNA together with 10 pM, 100 pM and 1 nM topotecan into apatite structure, cells were treated. Two days post treatment, MTT assay was conducted to produce results of cell viability (%) compared to untreated cells. ● CA/Topo, ● CA/siRNA/Topo.

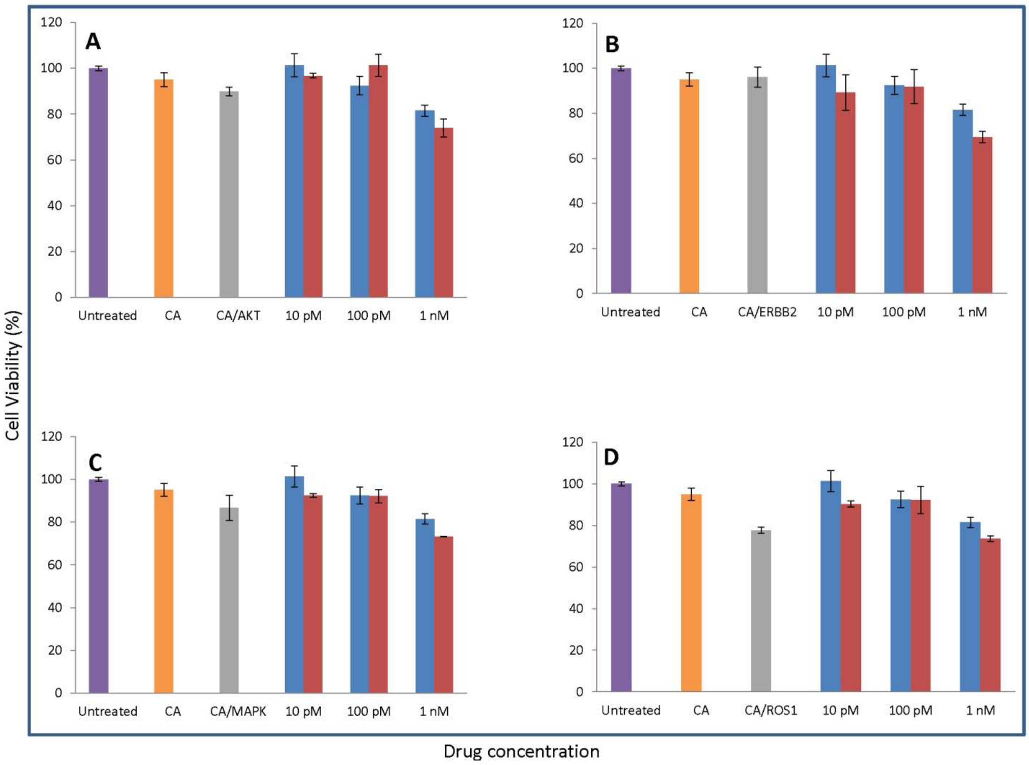

Figure S7. Effects of single pathway silencing on paclitaxel cytotoxicity in MDA-MB-231. Cells were treated with complexes of apatite formed with 3 mM of Ca together with 1 nM of AKT (**A**), ERBB2 (**B**), MAPK (**C**) and ROS1 (**D**) siRNA and 10 pM, 100 pM and 1 nM of Pac. MTT assay was performed two days after treatment and results were calculated as cell viability (%). ● CA/Pac, ● CA/siRNA/Pac.

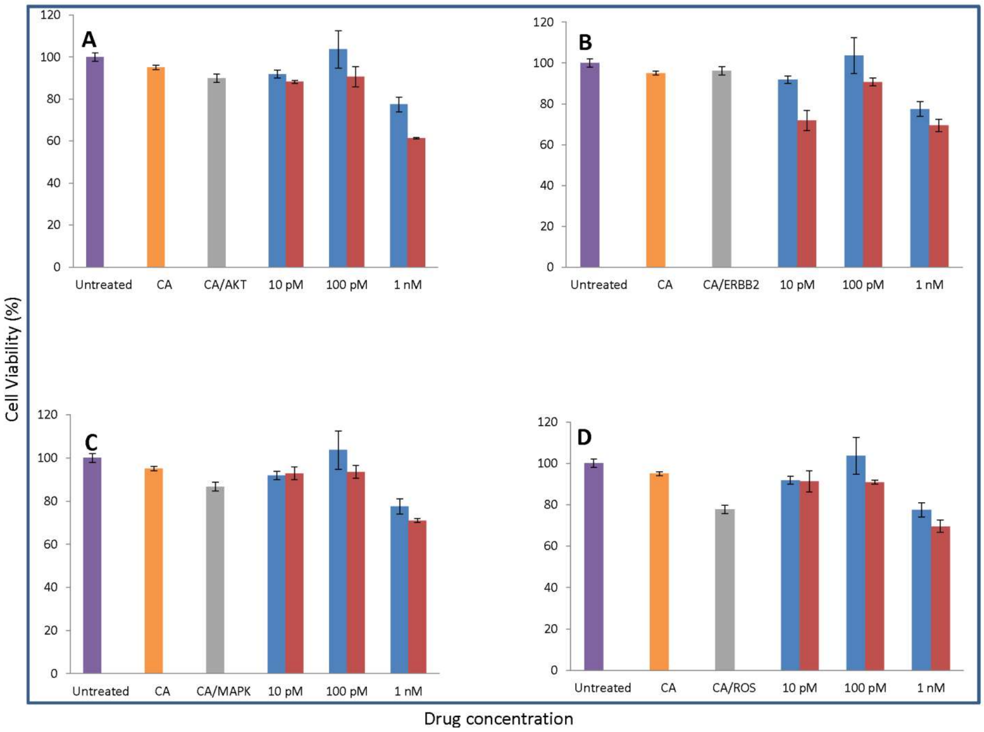

Figure S8. Effects of single pathway silencing on docetaxel cytotoxicity. MDA-MB-231 cells were treated with complexes of apatite formed with 3 mM of Ca together with 1 nM of AKT (**A**), ERBB2 (**B**), MAPK (**C**) and ROS1 (**D**) siRNA and 10 pM, 100 pM and 1 nM of Doc. MTT assay was performed two days after treatment and results were calculated as cell viability (%). ●CA/Doc, ● CA/siRNA/Doc.

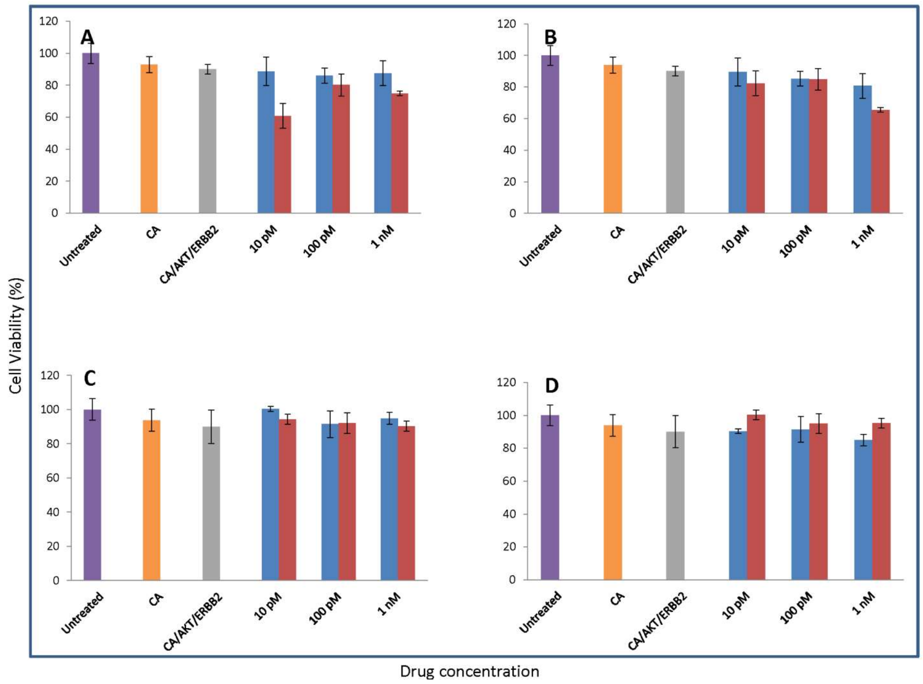

Figure S9. Effects of silencing AKT and ERBB2 oncogenes on drugs cytotoxicity in 4T1. Cells were treated with complexes of apatite formed with 3 mM of Ca together with 1 pM of AKT and ERBB2 siRNA and 10 pM, 100 pM and 1 nM of Pac (**A**), Doc (**B**), Mito (**C**), Topo (**D**). MTT assay was performed two days after treatment and results were calculated as cell viability (%). ● CA/drug, ● CA/AKT/ERBB2/drug.

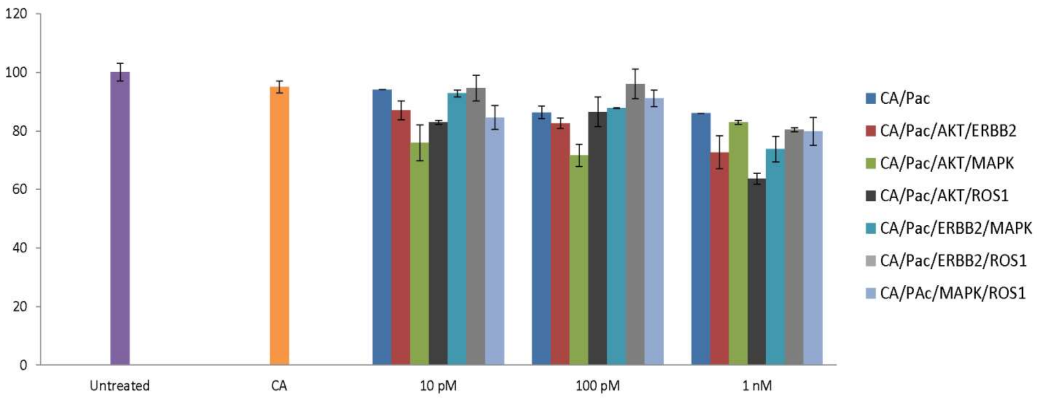

Figure S10. Cell viability assay on MDA-MB-231 cells treated with carbonate apatite complexed with paclitaxel and two siRNAs. By addition of 3 mM Ca, 1 nM siRNA and 10 pM, 100 pM and 1 nM of Pac, cells were treated and then subject to MTT assay after two days. Results are presented as cell viability (%).
